# What Is British National Insurance to Be?

**Published:** 1912-12-07

**Authors:** 


### The Hospital

The Workers' Newspaper of Administrative Medicine and Institutional Life, Administration, National Insurance and Health.

No. 1379, Vol. LIII. SATURDAY, DECEMBER 7, 1912. PRICE ONE PENNY.

### WHAT IS BRITISH NATIONAL INSURANCE TO BE?

### Less than Nothing for Men and Women Workers plus Degradation—or What?

From the outset we have assumed that the Chancellor of the Exchequer's intention was, through the National Insurance Act, to place the workers throughout the country, when seriously ill, in a safer, better, and more independent position in return for their compulsory payments weekly and those of their employers. On introducing the Bill in the House of Commons, Mr. Lloyd George laid it down that "it would provide free medical relief for every insured person with no taint of charity." This and many other statements made by Mr. Lloyd George have been accepted in good faith throughout the country, and we have tried to help the Government, through the voluntary hospitals, to maintain and enforce the principles represented by Mr. Lloyd George's words, which every impartial person has accepted in good faith as honestly meant and literally true.

The letter we publish in another column, from the Vice-Chairman of the Warwickshire Insurance Committee, implies a doubt as to the seriousness of the Chancellor's contentions. It may be well, therefore, to inquire what will be the exact position of insured persons should the Government repudiate the plain interpretation which has been put upon the assurances given by Mr. Lloyd George in his speeches and by Mr. Masterman in reply to questions in the House of Commons. It seems to plain men to be unthinkable that, despite the compulsory payments made under the National Insurance Act by men and women workers all over the country, their position in fact when ill and that of the voluntary hospitals will be materially worse than it was before Mr. Lloyd George intervened by an Act of Parliament. We, therefore, take the opportunity afforded by the delay in the publication of the results of the conference between the Chancellor of the Exchequer and the medical profession to try and elucidate the matter. A reference to page 273 will show the latest position taken up by Mr. Masterman on behalf of the Government on the question of business arrangements entered into between hospitals and approved societies. If those answers are to be taken seriously they clearly imply that the *pro rata* system can be adopted under the National Insurance Act. If so, the solution of the difficulties presented by the pressing need which will arise for hospital benefit for insured persons can be readily supplied. But Mr. Francis, the Vice-Chairman of the Warwickshire Insurance Committee, clearly holds that the Act provides no money and authorises no arrangements to be made as Mr. Masterman implies, and friends of the Government are declaring that the whole purpose of the National Insurance Act and its very restricted and small medical benefits have not yet been apprehended or understood by the insured, the approved societies, or the voluntary hospitals.

The Government's strictly legal attitude in relation to medical treatment for the insured is declared to be decisive. It is that, although 7d. a week is compulsorily paid by insured persons and their employers, it will not affect the former's position as objects and recipients of charity, *vis-à-vis* with the voluntary hospitals. In other words, insured persons will find that these payments and their position as insured persons entitle them in no sense to medical treatment equal to that provided for an in-patient, or even for an out-patient in any of the special departments, at the voluntary hospitals. True, insured persons will have compulsorily paid, if women 3d., and if men 4d. a week, for medical and sickness benefit, but these payments do not alter or improve their position as sick persons, compared with what it was before they made these contributions. On the contrary, the way in which the Act is framed, and the strict limitations imposed upon the character and quality of the medical treatment under the Act, will make insured persons, if anything, more than ever dependent upon charity for effective medical and surgical treatment whenever they may be seriously ill. Plain men, being Englishmen at any rate who are accustomed in their dealings with others to have regard to a standard of personal honour, and to expect and receive it from those with whom they deal, cannot believe that so scurvy a contract as that indicated by some of their supporters can in fact be true. If it is true, then, instead of getting 9d. for 4d., as Mr. Lloyd George proudly proclaimed, insured persons will in fact receive for their payments less or only the medical benefit they formerly enjoyed, plus the degradation of being officially stamped as dependants upon charity when seriously ill. To argue, as the Government must argue should they officially take up the attitude ascribed to them, means that they must contend that an insured person must also be regarded as a necessitous person when overtaken by grievous illness, and that no importance is attached to the spirit of thrift or independence which for generations has been regarded as the birthright and proud possession of British workers of all classes. The insured will be plainly told that their payments under the National Insurance Act will provide them with absolutely nothing as a right in the way of medical benefits, except only when suffering from minor ailments which can be treated at home. Whenever grave illness incapacitates an insured person from work and threatens his or her very life, the National Insurance Act, thus interpreted, makes the Government, like the Levite, pass insured persons by on the other side and leave them to seek relief from charitable institutions. If they fail of success, insured persons must go without adequate treatment, whatever dire consequences may result to themselves, and whatever miseries may be cast upon their families.

Again, assuming that the supporters of the Government are correct in maintaining the Government's intention to extend this merciless treatment to our British insured workers in the days of peril from illness or accident, what will be the position of the voluntary hospitals? The Government, having for strictly legal reasons ranged themselves as the modern exponents of the policy of the Levite in the parable, can the voluntary hospitals step in as the Good Samaritan and decide to treat all insured persons as objects of charity, on the official declaration of Mr. Lloyd George and the Government that, despite the National Insurance Act and the 15,000,000 men and women who have paid under it, not a single one of them is in any sense entitled to exercise a spirit of independence, but must with humility consent (to use the German artisan's term) to accept the degradation of charity, or to suffer, and possibly to die, without adequate medical and surgical treatment? No doubt the voluntary hospitals, if their resources were unlimited and assured, would gladly admit to treatment all applicants when grievously ill. But the managers in this matter of insured persons are not free agents. They are dependent even in the Metropolis to the extent of 50 per cent., excluding legacies, upon the voluntary contributions of benevolent people. In Scotland 62 per cent. of the voluntary income comes from the same source, excluding legacies, which amount to about a third of the gross revenue, excluding large donations for special purposes. In the provinces 68 per cent. of the ordinary revenue comes from benevolent donors, exclusive of legacies.

We have shown that the number of insured persons at present under treatment in the voluntary hospitals throughout the country varies from 50 per cent. in London to 60 per cent. or more in the provinces and Scotland. Should the voluntary supporters of hospitals who provide the money for the maintenance of the existing beds, on the ground that they are compulsorily compelled under the Insurance Act to contribute weekly payments for every person in their employ, withdraw their subscriptions, the effect must be that at least half the existing number of beds in the voluntary hospitals will have to be closed. We have throughout shown that the in-patient requirements of insured persons, if they are to be met, must continuously have the use of more than half the whole number of beds at present contained in the voluntary hospitals. Indeed in some large cities, and in the case of a good many hospitals, the whole of the existing hospital beds available may not prove sufficient to meet these requirements. The mere statement of plain facts like these, having regard to the conduct and statesmanlike action with which all former British Governments have safeguarded the interests of the people in these islands, must make every thoughtful person, quite apart from politics, hope that Mr. Lloyd George and the present Government will decline to act like the Levite in the parable.

We have knowledge that every member of Parliament has been made fully aware of the crisis ahead with which British workers throughout the United Kingdom are threatened through National Insurance, unless the representatives of these classes all over the country, with the co-operation of every benevolent and statesmanlike man in the House of Commons, step into the breach. We have shown that it is perfectly easy at a relatively small cost, which it is not difficult to provide, to give every British like every German worker as a right all the medical and surgical treatment which he may need. We will not, in view of the facts just brought out, believe that any British Chancellor of the Exchequer (who history records has heretofore been a gentleman of knowledgeable experience, zealous for the upholding of everything that is greatest and best in national affairs, wise and prudent in counsel) will stand before the nation as the upholder of the cruel and scurvy treatment of all British workers which is attributed to Mr. Lloyd George, until we receive an official notification, duly authenticated, that such is the case. Still it is imperative to commend, the issues here raised at this crisis in our national history, to the candid and impartial consideration of the press throughout the country, and of every member of the House of Commons as a man and a gentleman. Let Parliament forthwith lay or reveal this Levite policy.

